# Supplementary material for: Social context in political stance detection: Impact and extrapolation
Source: PLoS One. 2025 Jun 26;20(6):e0324697. doi: 10.1371/journal.pone.0324697 (PMC12201627; doi:10.1371/journal.pone.0324697)
Supplement: S1 — Confidence bands for the Macro F-1 score and Area Under the ROC curve of the within-country and out-of-sample cross-country predictions at different context levels. The confidence bands are computed from the corresponding percentiles of the 1000 bootstrapped samples constructed for each experiment (PDF) [file pone.0324697.s001.pdf]

Supporting Information: Confidence Bands

In this appendix we present the boostrapped confidence bands based on 1000 samples of the corresponding evaluation dataset at the main-country level and the different robustness experiments conducted.

Level		Model	Chile (%) Acc.	Chile (%) M-F1	Chile (%) ROC AUC	Bolivia (%) Acc.	Bolivia (%) M-F1	Bolivia (%) ROC AUC	Ecuador (%) Acc.	Ecuador (%) M-F1	Ecuador (%) ROC AUC	Colombia (%) Acc.	Colombia (%) M-F1	Colombia (%) ROC AUC
**Tweet Level**		Weak-Labeled Tweets	84.97-86.18	83.06-85	84.77-86.68	82.82-84.89	72.89-75.03	87.42-89.57	76.28-78.9	75.18-78.19	78.16-81.51	81.6-83.76	81.01-83.66	81.76-84.53
		All Original Tweets	75.15-75.95	74.77-76.36	75.71-77.04	71.28-73.04	66.08-67.85	76.93-78.05	69.14-71.07	64.54-66.79	71.79-73.53	72.58-74.6	69.7-72.7	83.17-84.86
**User Level**	Avg. Tw-Level	All Original	85.56-86.60	75.14-76.80	85.36-87.06	78.61-80.46	78.24-80.10	82.97-84.89	80.26-82.55	77.09-79.75	82.25-85.23	79.85-81.70	72.54-74.85	80.00-82.48
		With Retweets	94.44-95.11	87.98-89.39	95.20-96.32	88.28-89.74	88.22-89.70	95.21-96.21	91.49-93.07	89.59-91.53	94.99-96.51	80.54-82.33	66.29-68.99	92.27-93.80
		Retweet-BERT	92.45-93.20	82.52-84.29	91.93-93.28	74.73-76.68	74.73-76.68	83.18-84.97	70.53-73.05	61.50-64.54	72.32-75.14	78.30-80.21	71.22-73.67	78.79-81.30
		Transformer	95.73-96.23	91.01-92.08	97.39-98.01	93.58-94.48	93.58-94.48	**96.24-96.97**	94.45-95.48	93.74-94.90	**97.81-98.41**	**95.20-95.99**	93.71-94.75	97.99-98.49
**Network Level**	Homog.	TSPA	-	-	-	**94.58-95.42**	**94.58-95.42**	95.10-95.97	**95.40-96.32**	**94.79-95.84**	96.36-97.37	**95.97-96.71**	94.69-95.63	93.63-94.94
		Response	95.30-95.89	89.90-91.21	97.31-98.02	93.45-94.53	93.37-94.47	**96.41-97.24**	93.51-94.88	92.06-93.71	97.15-98.10	94.65-95.60	92.95-94.23	97.92-98.51
		Retweet	95.99-96.46	91.01-92.06	97.46-98.06	93.54-94.44	93.54-94.44	**96.20-96.92**	94.50-95.51	93.77-94.93	**97.83-98.44**	95.28-96.07	93.86-94.93	**98.20-98.67**
		Combined Network	95.47-95.98	89.38-90.61	97.37-98.00	93.59-94.50	93.59-94.50	**96.23-96.94**	94.11-95.18	93.37-94.60	**97.83-98.44**	95.22-96.01	93.71-94.75	**98.23-98.69**
		**Heterogeneous**	**97.18-97.61**	**93.90-94.86**	**98.39-98.82**	**95.38-96.15**	**95.38-96.15**	**96.66-97.36**	**95.76-96.65**	**95.25-96.23**	**98.37-98.89**	**96.65-97.34**	**95.64-96.51**	**98.64-99.15**

Table 1. 95% Confidence bands for the performance metrics of in-country Stance Classifiers at different context levels. Model performance metrics correspond to the 2.5 and 97.5 percentiles of 1000 bootstrapped samples of the test set. For each metric, the classifiers whose performance is not statistically different, at a 95% confidence level, from the best-performing model are highlighted in bold.

		Chile		Bolivia		Ecuador		Colombia	
		M-F1	ROC AUC	M-F1	ROC AUC	M-F1	ROC AUC	M-F1	ROC AUC
**Chile**	Avg. Tweet-Level (WR)	-	-	37.06-37.68	19.14-20.28	56.90-58.45	87.56-88.52	89.57-90.27	95.70-96.23
	User-Level Transformer	-	-	35.29-36.15	26.79-27.73	80.80-81.67	88.26-89.03	84.00-84.64	93.09-93.51
	**Hetero. Network-Level**	-	-	**22.40-23.19**	**17.92-18.78**	**88.60-89.27**	**97.04-97.40**	**92.31-92.82**	**98.36-98.62**
**Bolivia**	Avg. Tweet-Level (WR)	9.68-10.07	11.42-12.18	-	-	26.36-27.32	26.90-28.40	17.04-17.69	14.33-15.24
	User-Level Transformer	9.79-10.11	18.30-19.22	-	-	**24.93-25.58**	44.66-46.18	18.05-18.67	23.50-24.64
	**Hetero. Network-Level**	**3.03-3.22**	**7.18-7.88**	-	-	**25.45-26.06**	**24.32-25.47**	**5.21-5.58**	**9.12-9.95**
**Ecuador**	Avg. Tweet-Level (WR)	57.73-58.61	93.37-93.89	33.57-34.12	12.79-13.50	-	-	79.52-80.50	85.97-86.90
	User-Level Transformer	80.08-80.76	90.62-91.13	23.23-23.87	15.61-16.27	-	-	77.51-78.24	89.45-90.04
	**Hetero. Network-Level**	**91.92-92.33**	**97.01-97.31**	**9.57-10.04**	**4.88-5.27**	-	-	**95.30-95.68**	**98.18-98.46**
**Colombia**	Avg. Tweet-Level (WR)	80.54-81.23	91.61-92.26	**38.82-39.28**	38.28-39.34	45.15-46.22	79.64-80.84	-	-
	User-Level Transformer	73.18-73.78	93.02-93.46	**38.20-38.98**	29.46-30.34	**77.20-78.02**	85.88-86.66	-	-
	**Hetero. Network-Level**	**85.31-85.87**	**96.34-96.64**	42.03-42.75	**27.06-27.93**	72.79-73.68	**92.64-93.15**	-	-

Table 2. 99% Confidence bands for the Macro F-1 (%) score and Area Under de ROC curve of the out-of-sample cross-country predictions for classifiers at different context levels. Model performance metrics correspond to the 0.5 and 99.5 percentiles of 1000 bootstrapped samples from the column-country. This excluded users seen during the training of the country classifier (row). For each metric, the classifiers whose performance is not statistically different from the best-performing model, at a 99% confidence level, are highlighted in bold. For the Bolivian case study, the worst-performing classifier is highlighted.

			Accuracy (%)		M-F1 (%)		ROC AUC	
			New Users	Protests User	New Users	Protests User	New Users	Protests User
**Tweet-Level**	Weak-Labeled Tweets		68.82-70.58	69.86-71.55	60.75-62.5	69.17-71.03	78.05-79.91	78.12-79.96
	All Original Tweets		63.71-65.16	70.26-71.33	62.91-64.36	67.01-68.35	72.28-72.93	74.31-74.85
**User-Level**	Avg. Tweet-Level	All Original	76.58-78.34	80.76-82.45	76.30-78.05	78.78-80.64	81.97-83.83	83.20-85.04
		With Retweets	80.05-81.50	88.09-89.16	80.05-81.50	85.31-86.65	96.81-97.46	97.60-98.14
	Transformer		88.88-90.02	90.72-91.66	88.63-89.80	89.74-90.74	95.68-96.26	96.24-96.87
**Network-Level**	Homogeneous	Response	75.36-76.75	84.14-85.39	75.35-76.74	80.49-81.99	92.88-93.78	93.18-94.11
		Retweet	79.40-80.86	87.00-88.21	79.40-80.85	84.33-85.77	95.13-95.84	96.27-96.89
		Combined Network	71.09-72.59	81.54-82.86	70.93-72.43	76.60-78.29	90.46-91.52	91.10-92.16
	**Heterogeneous**		**98.60-98.95**	**99.29-99.55**	**98.55-98.92**	**99.21-99.50**	**99.77-99.91**	**99.75-99.93**

Table 3. 99% Confidence bands for the Out of sample Predictions of the Chilean Referendum at different context levels. These correspond to the classifier trained on the 2019 Chilean Protest Data, but with inverted labels (“Pro” government is considered “Against” the referendum and vice-versa). Model performance metrics correspond to the 0.5 and 99.5 percentiles of 1000 bootstrapped samples of the Referendum collection, disaggregated by whether a user was seen during the training of the Chilean Protest classifier. For each metric, the classifiers whose performance is not statistically different from the best-performing model, at a 99% confidence level, are highlighted in bold.
