# Supplementary material for: Social context in political stance detection: Impact and extrapolation
Source: PLoS One. 2025 Jun 26;20(6):e0324697. doi: 10.1371/journal.pone.0324697 (PMC12201627; doi:10.1371/journal.pone.0324697)
Supplement: Attachment — Submitted filename: Rebuttal_Letter.pdf [file pone.0324697.s002.pdf]

Dear Reviewers and Editor,

We thank you for your thorough and helpful comments. We have attempted to address each; the comments have greatly improved the quality of the paper. Below we outline how we attempted to address comment point-by-point:

EDITOR COMMENTS

1. Please ensure that your manuscript meets PLOS ONE's style requirements, including those for file naming.

Corrected the title so it is in compliance with the style requirements.

2. We note that the grant information you provided in the 'Funding Information' and 'Financial Disclosure' sections do not match.

When you resubmit, please ensure that you provide the correct grant numbers for the awards you received for your study in the 'Funding Information' section.

Ensured the funding information in the Financial Disclosure sections is correct

3. We note that you have provided funding information that is not currently declared in your Funding Statement. However, funding information should not appear in the Acknowledgments section or other areas of your manuscript. We will only publish funding information present in the Funding Statement section of the online submission form.
   Please remove any funding-related text from the manuscript and let us know how you would like to update your Funding Statement.

We removed all funding information from the 'Acknowledgments' section and updated it accordingly.

4. When completing the data availability statement of the submission form, you indicated that you will make your data available on acceptance. We strongly recommend all authors decide on a data sharing plan before acceptance, as the process can be lengthy and hold up publication timelines.

We comply with the suggestion and make all data publicly available before acceptance. The data and code can be accessed through the following URLs:
https://doi.org/10.5281/zenodo.14207926
https://huggingface.co/Ramavill/twBETO_v0
https://github.com/rvillaco/Protest_Stance_Detection

5. Please include captions for your Supporting Information files at the end of your manuscript, and update any in-text citations to match accordingly. Please see our Supporting Information guidelines for more information: http://journals.plos.org/plosone/s/supporting-information.

We removed all supporting information files from the submission as they are now freely accessible through the URLs mentioned above.

REVIEWER 1 RESPONSES

C1. Current title is "Evaluating the Effect of Heterogeneous User Interactions in Out-of-Sample Stance Classification".
C1a. The use of "Stance Classification" is too general, and it may give the impression that the model can be applied to any stance detection task. Examples where it doesn't apply: detect if the author agrees or not with a previous comment; query if the author agrees with a given statement "X" --> Recommendation: use "Political Stance Detection".

C1b. Unclear what is meant by "effect of heterogeneous user interactions". The effect being evaluated is the **impact** on Political Stance Detection Performance. What is being evaluated is the **impact** of **accounting for**. It is hard to understand the meaning of 'heterogeneous' directly from the title.

We agree that the title was overly-general and appreciate the comments. We updated the title to “Social context in political stance detection: Impact and extrapolation”

C2. Given that there is no evidence that the user sample is representative of Chile's population, the fact that it is closer to the final vote cannot be used to claim that the proposed model is good. The correct way of doing this is by performing demographic inference and reweighing samples accordingly (see ref below). Authors could attempt to do this based on the author names and description with the due ethical considerations. Zijian Wang, Scott Hale, David Ifeoluwa Adelani, Przemyslaw Grabowicz, Timo Hartman, Fabian Flöck, and David Jurgens. 2019. Demographic Inference and Representative Population Estimates from Multilingual Social Media Data. In The World Wide Web Conference (WWW '19). Association for Computing Machinery, New York, NY, USA, 2056–2067. https://doi.org/10.1145/3308558.3313684
Recommendation: either follow the work above to apply the correct weighting OR downplay the fact that the proposal model is close to the final vote and add this disclaimer.

We agree with the observation and attempted to reweight the observations according to estimated demographic information. However, given the size of the referendum collection, the profile pictures were not saved when the collection took place. We attempted to download the pictures based on the saved JSONs but a large percentage of the user profile links were no longer available. Moreover, given the number of users (over 100k) and the new API rate limits imposed after the change from Twitter to X, it was not viable to attempt to download the profile

pictures of the accounts that are still active (it is likely a considerable number of accounts no longer exist).

We tried to estimate the demographic information based on only on text features (replacing the pictures with random noise as provided in the paper's repository) but the performance of the prediction dropped considerably below the ablation results provided in the paper (gender macro F1 dropped by 20%). We measured this based on a small sample of 50 accounts of politicians, reporters and institutions we constructed to test the method. The authors note that "We recommend using image data whenever possible to get the most accurate predictions", but we hypothesize the larger drop is also probably caused by the difference between European and Latin American Spanish. We also observed a drop in performance, as discussed in our paper, when comparing TWilBert (a model trained in European Spanish) with TwBETO (the model released in this paper).

Given these caveats, we decided to downplay the fact that the heterogeneous network model is closer to the final vote and added the disclaimer as recommended. This was done by removing the mention of this validation from the introduction, discussing this limitation in the "Robustness over time" subsection, and adding the disclaimer and reference to the reweighting approach in the limitation section.

C3. F1 scores are a function of the decision threshold. The optimal decision threshold varies based on the predicted scores distribution, hence it changes across datasets.
Recommendation: Authors should opt for metrics that are robust to the differences between domains, such as AUC or area under the precision-recall curve (AUPR).

Agreed, we included ROC AUC metric to all tables and analyzed it accordingly.

C4. The manuscript states in l. 94 that:
"Even though there exists work that has explored user-level stance detection on Twitter data [15], the proposed approach requires pairwise similarity calculations for all users of interest, which limits the scalability of the approach" Looking at Darwish et al. 2020, it seems that their algorithm depends on UMAP for dimensionality reduction and DBSCAN for clustering. Both algorithms have hyperparameters that allow them to scale by considering a smaller number of neighbors.

It is not clear that the algorithm cannot run on the present data. In fact, Darwish et al. have applied it to datasets of 1.8M, 2.4M and two datasets of 2.6M tweets, which are in the same scale as the datasets in the manuscript.

We have added additional clarification on this point; while Darwish et al. have datasets with millions of tweets, they only ran their approach on a maximum of 5,000 users (Table 2 in their paper). As the authors outline in the Finding Stance Clusters section, they calculate three pairwise cosine matrices for every user in their selected subset and run umap on their pairwise cosine similarity matrices. For the users in Chile alone, generating a single cosine similarity matrix for all of our users using their approach was set to take over 100 days. We added an additional sentence about the cosine similarity bottleneck in the Related Work section.

C5. Abstract refers to "users' social networks" which is a bit ambiguous. To distinguish from the platforms, authors should consider using the term 'egonets'.

Agreed, we updated the abstract accordingly and other references found in the main text that might lead to a similar confusion.

C6. To deal with class imbalance, authors use resampling, which tends to leave out some examples from the majority class. Have you consider using class weights in the loss function?

We did try weighting observations by the inverse of their class frequency and also other static resampling approaches, but found that dynamic resampling improved training stability as mentioned in the paper. We hypothesize that this is in part due to the dynamic sampling of n_max tweets for each user at the start of a batch, which implies that even repeated users have a good chance of including different tweets when added to the batch. At least 83% of the users in each training set had 15 or more tweets in the dataset. We added this discussion as it was not mentioned before.

Minor comments
l. 474 Undefined reference to Table 2.

Corrected broken reference.

REVIEWER 2 RESPONSES

Weakness: Some issues regarding the impact of each model component and the methodology to train and evaluate them must be clarified. First, the social network component and its emerging communities may alone answer the problem of user stance in favor or against protests. Second, the labeling process for training the model is superficially explained to make a self-contained manuscript.

On the first point, we agree that the network alone carries significant signal, and have added additional language highlighting that the TSPA baseline serves as a network baseline without any account features. TSPA's performance is surprisingly strong even without user text attributes, although the User Transformer outperforms it in some countries (when considering the ROC AUC performance). We added language further emphasizing that point in the Methods and Results sections. We further expand this point in the responses below.

On the second point, we added further description of the labeling methodology. See responses to the data description comments for further details.

1 Introduction: Regarding the first contribution, I would recommend that the authors also mention the impact of each compartment of the proposed architecture on the stance-detection problem. As reported in the following comments, it is important to justify the construction of an architecture based on three compartments (tweet, user, and network layers), given its high computational cost.
We added more details justifying this architecture choice in the introduction, as it allows us to evaluate the effect of context on the in-sample performance and generalization (this is

highlighted across the first 3 contributions). Other details on how this effect is manifested are addressed in the points discussed below and in the original results section.

2. Related Work: I recommend explaining specific technical terms such as “hand-labeled” and “hand-crafted” to make the manuscript clear for readers from broad research areas.
Added further description to clarify the aforementioned terms.

3. Data Description

Authors must provide further information about the adopted methodology to construct a ground truth dataset of labeled comments and evaluate the proposed framework. First of all, the weak-labeling step of the methodology must be explained in order to produce a self-contained manuscript. Questions that are raised from the current data description are:
- How is the “endorsement of hand-labeled political figures” leveraged?
The methodology relies on the hypothesis that users are more likely to tweet (or retweet) stance-tags (hashtag campaigns with well-defined stances towards each government and that occur at the end of a tweet) or political figures that are aligned with their stances during these events. For this reason, stance labels are assigned to a user if the percentage of tweets with a consistent stance-tag or retweets from political figures with a consistent stance is above a given threshold (the authors use 90% as a threshold based on a labeled validation set). A final stance label is assigned to a user if the stance obtained by both signals (usage of hashtags and retweet of political figures) is consistent. This discussion was added to the paper.

- Which hashtag campaigns have well-defined stances toward each government? I recommend providing a table with prominent examples.
Added a table with prominent examples.

- Which polarized communities are observed according to user partitions from the constructed labels? It appears a network of users supports the weak-labeling methodology, but no information is available on how this network and connections between users are constructed.
The authors validate the quality of the weakly-annotated labels based on a hand-labeled sample of users and by showing that the constructed labels partition the users in communities that are polarized in their language and news-sharing behavior in a way consistent with the ideological underpinnings of each protest. These communities are not explicitly defined, but are shown to use semantically consistent text and expressions by leveraging an established machine translation algorithm. They show that terms related to left-leaning ideologies in one community tend to be discussed in similar contexts as right-leaning terms (e.g., Socialism mistranslates to Fascism); terms related to law and order in one group are discussed in a similar context as the other discusses oppression, or that opposition leaders are discussed in similar contexts as government representatives. This discussion was added with examples of how this polarization is manifested in the data description section.

The observation of polarized communities raises the issue of whether the users' network and its emerging communities alone answer the problem of user stance in favor or against protests. What is the relevance (i.e., the impact) of the users' text content for detecting their instances? This issue should be addressed in the analyses, and the impact of each component on the stance problem in the context should be clarified.
Added a brief discussion of this objective in the data section and explore it in more detail in the results section as described in the responses below.

Why the weak-labelling is not used for the Chilean Referendum dataset?
As discussed in the "The 2020 Chilean Plebiscite 224" subsection, we applied the same weak-labeling methodology, based on two stance signals, to construct the referendum dataset. This section also provides further details on the weak-labeling methodology used for the construction of the protest dataset, addressing in part the previous comment and making the paper more self-contained (given that we follow the same algorithm).
However, as mentioned, the difference lies in that instead of using hand-labeled Political Figures, as was done to construct the weak-labels for the South American protests, we opted to identify the hashtags used in the user description that were explicitly rejecting or approving the referendum in first person. This simplified the labeling procedure given that we labeled other stance tags for the referendum. We found that the usage of stance tags in the descriptions as a replacement signal also resulted in meaningful polarized stance partitions based on the same language metrics used to validate the protest weak-labels (we did not explore polarization in news-sharing behavior during the referendum as it escaped the scope of this work). This discussion was added to the paper to clarify this topic.

4. Materials and Methods

Some notes and recommendations for better comprehension of Fig1:
- Include input arrows for each layer to indicate where to start reading each layer, mainly the first and second ones, which have more details.
Added a white arrow to denote the first input to each component. Other than this, the figure already included arrows that showed the flow through the architecture. However, if the white arrow for the last component adds too much clutter (as all input arrows point to the center), we can remove it. Finally, to further explain the last component, we added how the output of the user transformer is used to get the input embeddings of a user and her ego-network. We also added an arrow to the corresponding classification head.

- Should the output dimension of the first layer (tweet encoder) be equal to the input dimension of the second layer (user encoder)? Please check it and explain it in the text.
No, the input of the second layer adds a [CLS] vector (of the same embedding of a tweet encoded by the first layer). For this reason, the input for the second layer is (b_size, n_max+1, h_hid). This was described in the text of the paper, but to further clarify we added this to the figure explicitly and discussed it in the caption of the figure.

- The Lx label's current position in the figure is difficult to understand. It must be checked.

Centered Lx and added brackets so that it is clearer that the parameter refers to the stack of transformer layers.

- Labels in the third layer (network) must be explained.

Added description of the parameters to the caption and, as mentioned before, added user embedding vectors to clarify the relationship with the previous layer.

- The quality of Figure 1, shown on page 26 of the reviewer file, is very bad. It is important to fix it.

Recreated the figure ensuring that is 600dpi.

Section 4.1: The dimensions of the user tweets input and the tweet embeddings output are not explained in the text. Please explain it.

Added further description of the dimensions to the figure's caption and added to the main text the size of the hidden dimension maintained throughout all components (768).

5. Results

Regarding the results shown in Table 3, are the performances of the proposed User-Level Transformer and TSPA approach significantly different?

For all countries, the performance of the User-Level Transformer and TSPA are significantly different based on boostrapped confidence intervals (see the answer to next point). Interestingly, even though TSPA tends to produce better F1 scores, its ROC AUC is lower than the User-Transformer. This discussion was added to the paper.

A confidence interval for the performance metrics could be calculated to analyze whether they are significantly different. Approaches such as Normal Approximation Interval or Bootstrapping the Test Sets are currently good practices.

Agreed, we recomputed the model performance metrics based on 1000 bootstrapped samples of the test set (for the robustness analysis this corresponds to the column country or the referendum data as explained in each table's caption). The values presented in the tables changed slightly as we now report the median result. For each metric, the classifiers whose performance is not statistically different, at a given confidence level (95 for the main country and 99 for the robustness analysis), from the best-performing model are highlighted in bold. The corresponding confidence bands are included as an appendix to avoid clutter, but if deemed important, they could be moved to the main body of the paper.

The network-level appears to improve the results significantly. Again, communities that emerge from the network, which were used to label users, are probably the most relevant information for user stance in favor or against protests. Thus, the result in Table 3 is the expected one (i.e., no novelties). Authors should discuss, based on their data, the relevance of exploring text content once you have the users-network, which is probably the most relevant information for stance in the analyzed context.

We added further discussion in methods of how TSPA serves as a network-only baseline. We show that while TSPA achieves strong performance, it does not outperform our heterogeneous models. Moreover, when comparing their performance based on their ROC AUC, the User Transformer is significantly better. This discussion is added to the paper.

The results shown in Section 5.2 are very interesting. Specifically, the case of Bolivia shows that models trained with data from a given location cannot always be applied to other locations, e.g., countries, even though they share similar cultures and languages. On the other hand, the issue discussed above becomes more evident as cross-country results show that the users' network is the information that allows models to reach the best results (e.g., accuracy and F1 greater than 90% in most evaluated cases). Again, it is important to discuss the relevance of exploring text content once you have the users' network, considering the computing cost to deal with each one.

Added additional TSPA context and, during the analysis of the Bolivian adversarial setting stress that “This is consistent with the observed tendency of deep learning models to be overconfident in their predictions and highlights the importance of domain knowledge when applying these models to different domains.”

However, we stress that the homogenous network models can also significantly underperform models like the User-transformer that only leverage semantic features. The following discussion was added to the referendum results section as it was not sufficiently elaborated on before: “We hypothesize that this might be due in part to differences in network construction: the Referendum data included the timeline of all users collected, whereas this was not done for the country-level protest data. This implies that the networks constructed for the former dataset are denser and include more reply and quote interactions between users. Hence, it more likely that we capture both positive and negative responses from one user to others of the same or different stances. While the User-level transformer can leverage the semantic context of each tweet as it pertains to the stance of the user, the homogenous models are forced to collapse these disparate signals into one interaction link. As such, we see that the Retweet-based homogenous model shows the least degradation (as retweets provide more consistent endorsements), while the combined network model drops significantly below other simple average user-level models. However, when the model is allowed to leverage the heterogeneous signals provided by retweets and other responses, its performance improves dramatically. Moreover, except for the heterogeneous network model, the performance of all classifiers is significantly reduced when dealing with completely new users at a 99% confidence level. This again highlights how the disparate signals present in a user's ego-network can improve the robustness of the classifier over time.”

To correct the broken reference for Table 5 on page 13/18, line 474.
Corrected broken reference to Table 2.
